# Associations of Anxiety and Depression and Hypomanic Traits with Self-Informant Discrepancies in Adults First Diagnosed with ADHD in Adulthood: A Cross-Sectional Study

**DOI:** 10.3390/jcm15103965

**Published:** 2026-05-21

**Authors:** Jiaqi Song, Yuanyuan Li, Jiajun Xu

**Affiliations:** Mental Health Center, West China Hospital, Sichuan University, No. 37 Guoxue Xiang, Chengdu 610041, China; songjiaqi2@stu.scu.edu.cn (J.S.); xujiajun120@126.com (J.X.)

**Keywords:** adult ADHD, self-informant discrepancy, anxiety, depression, hypomanic traits, DIVA-5

## Abstract

**Background/Objectives**: Adults first diagnosed with ADHD in adulthood are a clinically distinct subgroup, yet the factors associated with self-informant discrepancies in this group remain underexplored. This study examined the associations of anxiety, depression, and hypomanic traits with self-informant discrepancies on the DIVA-5. **Methods**: A total of 217 adults first diagnosed with ADHD in adulthood and their parents independently completed the DIVA-5. Patients also completed the Self-Rating Depression Scale (SDS), Self-Rating Anxiety Scale (SAS), and the 32-item Hypomania Checklist (HCL-32). Associations were examined using Spearman correlations, group comparisons, and hierarchical linear regression, controlling for demographic covariates. All reported *p*-values were adjusted using the Benjamini–Hochberg false discovery rate method. **Results**: Self-informant discrepancies were significant across all symptom and functional impairment domains, with self-ratings consistently higher than parent-ratings. Anxiety showed robust independent associations, particularly for hyperactivity-impulsivity (both childhood and adulthood) and childhood functional impairment. Depression showed positive associations only in separate hierarchical linear regression analyses for each affective characteristic; these associations were no longer significant after controlling for anxiety. Hypomanic traits were not independently associated with self-informant discrepancies in any domain. **Conclusions**: Among adults first diagnosed with ADHD in adulthood, anxiety was robustly associated with self-informant discrepancies, whereas depression and hypomanic traits were not. These findings underscore the importance of attending to anxiety when interpreting self-informant discrepancies in this population.

## 1. Introduction

Attention deficit hyperactivity disorder (ADHD) is a common neurodevelopmental disorder, with a global prevalence of approximately 2.5% among adults [1,2]. Adult ADHD is associated with significant occupational, interpersonal, and functional impairments [3], making its identification and management of considerable clinical importance. Epidemiological data further indicate that more than half of adults with ADHD are first diagnosed in adulthood [4], particularly among females [5].

The assessment of adult ADHD relies heavily on subjective reports of symptoms and functional impairments, as no objective biomarkers are currently available [6]. Clinical guidelines recommend integrating reports from both patients and informants (e.g., parents) to obtain more comprehensive information [6]. In practice, however, clinicians often find substantial discrepancies when integrating reports from different sources [7,8]. Prior research has found only low-to-moderate correlations between self- and informant-reports, with patients often reporting greater symptom severity than informants [9,10]. Such discrepancies pose considerable challenges for clinical decision-making. To explain the sources of these self-informant discrepancies, previous studies have proposed various hypotheses. Some studies suggest that implicit symptoms, such as inattention, are difficult for informants to observe, which may lead to lower ratings in informants’ assessments [11]. The positive illusion bias theory posits that patients may exhibit self-perception biases, tending to underestimate their own functional impairments [12]. Additionally, symptom exaggeration and malingering are also considered potential sources of self-informant discrepancies [13,14]. These hypotheses provide explanations from the perspectives of observation, self-perception, and reporting motivation, respectively.

Apart from the factors mentioned above, affective characteristics have been increasingly recognized as potential factors associated with self-informant discrepancies in adult ADHD [15,16]. In recent years, research has shown that elevated scores on self-report depression and anxiety scales can be useful in screening for possible symptom overreporting during ADHD evaluations [16]. Internalizing and somatic symptoms have also been associated with discrepancies between subjective cognitive complaints and objective performance [17]. Some studies have further explored potential underlying mechanisms: depression is associated with negative cognitive biases that may amplify attention to and appraisal of negative experiences [18], whereas anxiety is related to hypervigilance, which may lead to overestimation of symptom severity [19]. Furthermore, hypomanic traits may also be relevant to self-informant discrepancies. Previous research in non-ADHD populations has found that individuals with hypomanic traits exhibit positive cognitive biases [20], tending to overestimate positive traits when evaluating their own performance and even providing extreme self-assessments [20,21].

Although prior research has laid a foundation for understanding the association between affective characteristics and self-informant discrepancies in adult ADHD, some important questions remain unresolved. Existing studies have generally not distinguished between adults diagnosed with ADHD in adulthood and those diagnosed in childhood [8]. Adults first diagnosed with ADHD in adulthood differ from those diagnosed in childhood in several important respects. Studies indicate that this group tends to have higher educational attainment [22], presents more often with emotional difficulties than with overt behavioral problems [22], and consists predominantly of females [5]. Moreover, symptoms in this population are frequently more covert [23], and diagnosis is often delayed by complex compensatory strategies and masking behaviors [24,25,26]. Despite these distinctive clinical features, the association between affective characteristics and self-informant discrepancies in this specific subgroup has not yet been directly examined. In addition, research examining the association between hypomanic traits and self-informant discrepancies in adult ADHD remains limited [20,21]. Whether such traits relate to assessment discrepancies in this population remains unclear.

This study focuses on adults first diagnosed with ADHD in adulthood, examining the associations between affective characteristics (anxiety, depression and hypomanic traits) and self-informant discrepancies on the DIVA-5. It is important to note that the aim of this study is not to validate the diagnostic utility of the DIVA-5. Rather, we use the DIVA-5 as a standardized framework to quantify self-informant discrepancies regarding symptoms and functional impairment across different developmental periods.

## 2. Materials and Methods

### 2.1. Study Design and Ethics

This was a cross-sectional study. The research protocol was approved by the Ethics Committee of West China Hospital, Sichuan University (Approval No. 2025/2025) and conducted in accordance with the principles outlined in the Declaration of Helsinki. Written informed consent was obtained from all participants and their parents prior to participation.

### 2.2. Participants

A priori sample size calculation was performed using G-Power 3.1. Assuming an independent samples *t*-test with a medium effect size (Cohen’s d = 0.50), α = 0.05, and power (1 − β) = 0.90, the minimum required sample size was determined to be 86 participants.

Participants were adult ADHD patients recruited from outpatient clinics at West China Hospital, Sichuan University, between 23 November 2025, and 30 January 2026. The inclusion criteria were: (a) met DSM-5 diagnostic criteria for adult ADHD, confirmed by three psychiatrists with over ten years of clinical experience, ensuring diagnostic accuracy; (b) age between 18 and 60, and first diagnosed with ADHD at the time of study participation; (c) involvement of one parent in clinical interviews; (d) had not taken any ADHD medications at any time prior to or during study participation (to avoid potential drug effects). Exclusion criteria were: (a) concurrent severe physical or neurological disorders; (b) acute phase of other psychiatric disorders (e.g., schizophrenia, bipolar disorder), severe suicide risk, or acute substance abuse; (c) any condition that would impede the ability to comply with the study procedures.

A total of 217 adults with a first-time ADHD diagnosis in adulthood were included. During the assessment, the following demographic and background information was also collected: age, sex, education level, and the participating parent’s familiarity with the patient, rated by the parent as “less familiar,” “familiar,” or “very familiar.”

### 2.3. Clinical Assessment Instruments

#### 2.3.1. ADHD Symptom and Functional Impairment Assessment

The Diagnostic Interview for Adult ADHD (DIVA-5) is a semi-structured interview tool specifically designed for the assessment of adult ADHD based on DSM-5 diagnostic criteria. It comprises three core sections: inattentive symptoms, hyperactive-impulsive symptoms, and functional impairments, each evaluated separately for childhood (<12 years) and adulthood (the past six months). Previous studies have demonstrated DIVA-5’s good reliability and validity [27,28]. In this study, patients and one informant (parent) completed the DIVA-5 independently. All interviews were conducted by psychiatrists with at least ten years of clinical experience. To quantify the discrepancies between self-reports and parent reports, this study calculated the difference scores (self-report minus parent report) between patient-reports and parent-reports across the six domains of the DIVA-5, specifically: childhood inattention, childhood hyperactivity-impulsivity, childhood functional impairment, adult inattention, adult hyperactivity-impulsivity, and adult functional impairment. A positive score indicates that the self-report score is higher than the parent-report score. These difference scores were used as the dependent variables in all subsequent analyses examining associations with affective characteristics.

#### 2.3.2. Assessment of Affective Characteristics

The Self-Rating Depression Scale (SDS) was used to evaluate the severity of depressive symptoms over the past week. This 20-item scale uses a 1–4 rating scale, with scores converted to a standardized score (raw score × 1.25). Higher scores indicate greater depressive severity. According to the Chinese normative data, a standardized SDS score ≥ 53 is defined as meeting the clinical threshold for depressive symptoms. Severity is further categorized as follows: 53–62 points indicate mild depression, 63–72 points indicate moderate depression, and ≥73 points indicate severe depression. The Cronbach’s alpha coefficient for this scale is 0.849.

The Self-Rating Anxiety Scale (SAS) was used to assess the severity of anxiety symptoms experienced by patients over the past week. This 20-item scale uses a 1–4 rating scale, with scores converted to a standardized score (raw score × 1.25). Higher scores indicate greater anxiety severity. According to the Chinese normative data, a standardized SAS score ≥ 50 is defined as reaching the clinical threshold for anxiety symptoms. Severity is further categorized as follows: 50–59 points indicate mild anxiety, 60–69 points indicate moderate anxiety, and ≥70 points indicate severe anxiety. The Cronbach’s alpha coefficient for this scale is 0.835.

The 32-item Hypomania Checklist (HCL-32) was used to assess hypomanic traits. This 32-item questionnaire requires “yes” or “no” responses, with a total score ≥ 14 indicating high hypomanic traits. It should be noted that the HCL-32 assesses lifetime trait tendencies rather than current mood states [29]. The Cronbach’s alpha coefficient for this scale is 0.863.

It should be noted that the SAS and SDS scales in this study were used to assess recent emotional states, and the HCL-32 was used to assess hypomanic traits. None of these measures is equivalent to a clinical diagnosis. For brevity, terms such as “anxiety,” “depression,” “hypomanic traits,” or “mild/moderate/severe depression/anxiety” are used throughout the manuscript to refer to scale-based scores or derived groups (e.g., clinical threshold, severity levels), and should not be taken to indicate formal diagnostic status.

### 2.4. Statistical Analysis

All data were analyzed using SPSS version 26.0 (IBM Corp., Armonk, NY, USA). No missing data were present in any variables in this study, thus no missing value handling was required. Descriptive statistics were calculated for all variables. Intraclass correlation coefficients (ICC) with two-way mixed-effects models assessed agreement between self- and informant-reports for continuous scores. Paired *t*-tests and Wilcoxon signed-rank tests compared mean differences, with effect sizes calculated as Cohen’s d.

Associations between affective characteristics and discrepancies were examined using Spearman’s correlations, Mann–Whitney U tests (for HCL-32 groups), and Kruskal–Wallis H tests (for SDS/SAS severity grades). To assess independent associations while controlling for demographic variables (gender, age, education, parental familiarity), separate hierarchical linear regression analyses were conducted for each affective characteristic. In these models, demographic covariates were entered in Block 1, and each affective variable was entered individually in Block 2. Combined hierarchical regression analyses were further performed to evaluate the independent associations of anxiety, depression and hypomanic traits, with anxiety and depression entered in Block 2 and hypomanic traits entered in Block 3. To examine whether demographic characteristics moderated the observed associations, interaction terms (affective characteristic × gender, affective characteristic × education) were added to the combined models. For affective characteristics showing significant associations in the above analyses, additional hierarchical linear regression analyses were conducted with severity grades as the predictor. Standardized regression coefficients (β) were reported. Multicollinearity was assessed using variance inflation factors (VIF), with values < 5 considered acceptable. All reported *p*-values were adjusted using the Benjamini–Hochberg false discovery rate (FDR) method, with statistical significance set at corrected *p* < 0.05 (two-tailed).

## 3. Results

A total of 217 adult ADHD patients were included in this study. Demographic and clinical characteristics are shown in Table 1. The mean age of the participants was 24.48 years, predominantly female (72.8%). Most patients held a bachelor’s degree or higher (77.0%). Regarding affective characteristics, 71.9% of participants met the clinical threshold for depressive symptoms, 57.6% met the clinical threshold for anxiety symptoms, and 72.8% exhibited high hypomanic traits (HCL-32 ≥ 14). Additionally, 14.7% of parents reported being “less familiar” with patients. No significant differences in parent-reported scores were observed across different levels of parental familiarity (FDR-corrected *p* > 0.05).

### 3.1. Self-Informant Discrepancy Analysis

Significant discrepancies between patient reports and parent reports were observed across all symptom and functional impairment domains (Table 2).

Intraclass correlation coefficients (ICC) ranged from 0.062 to 0.290, indicating poor agreement overall. The highest agreement was observed for childhood hyperactivity/impulsivity (ICC = 0.290, 95% CI [0.027, 0.491]), while the lowest were found for adult functional impairment (ICC = 0.062, 95% CI [−0.043, 0.173]) and adult inattention (ICC = 0.066, 95% CI [−0.048, 0.190]), with confidence intervals containing zero.

Paired-sample *t*-tests revealed that self-reported scores were significantly higher than parent reports across all domains (all FDR-corrected *p* < 0.001), with effect sizes ranging from medium to large (Cohen’s d = 0.62–0.92). Wilcoxon signed-rank tests yielded similar results (all FDR-corrected *p* < 0.001). Examination of the direction of differences showed that for adulthood inattention, 84% of patients rated themselves higher than their parents, while only 5% showed the opposite pattern. For hyperactivity/impulsivity, the proportion of parents rating higher than patients (positive ranks) was relatively higher (11–12%).

### 3.2. Associations Between Affective Characteristics and Self-Informant Discrepancy

#### 3.2.1. Correlation Analyses

Spearman correlation analyses revealed distinct patterns for each affective characteristic (Table 3). SAS scores were positively correlated with discrepancies across all domains except childhood inattention (r_s_ = 0.154–0.282, all FDR-corrected *p* < 0.05), with the strongest correlation observed for childhood hyperactivity/impulsivity (r_s_ = 0.282, FDR-corrected *p* = 0.009). SDS scores were positively correlated with discrepancies across all domains except adult functional impairment (r_s_ = 0.150–0.233, all FDR-corrected *p* < 0.05). HCL-32 scores were correlated only with discrepancies on hyperactivity/impulsivity domains (childhood: r_s_ = 0.166, FDR-corrected *p* = 0.032; adult: r_s_ = 0.177, FDR-corrected *p* = 0.027).

Consistent findings emerged when examining clinical thresholds and severity grades (Table 4). Reaching the clinical threshold for anxiety (SAS ≥ 50) and anxiety severity were positively correlated with discrepancies on childhood hyperactivity/impulsivity and childhood functional impairment (r_s_ = 0.230–0.272, all FDR-corrected *p* < 0.05). Reaching the clinical threshold for depression (SDS ≥ 53), depression severity, and high hypomanic traits (HCL-32 ≥ 14) were not significantly correlated with discrepancies on any domain (FDR-corrected *p* > 0.05).

#### 3.2.2. Group Comparisons of Discrepancies Based on Affective Characteristics

Group comparisons further corroborated the above findings (Table 5). Patients meeting the clinical threshold for anxiety (SAS ≥ 50) exhibited higher discrepancies on childhood hyperactivity/impulsivity (FDR-corrected *p* = 0.024), adult hyperactivity/impulsivity (FDR-corrected *p* = 0.024), childhood functional impairment (FDR-corrected *p* = 0.024), and adult functional impairment (FDR-corrected *p* = 0.048) compared to those who did not meet the threshold. In contrast, no significant differences were found between patients meeting the clinical threshold for depression (SDS ≥ 53) and those below the threshold, nor between patients with high hypomanic traits (HCL-32 ≥ 14) and those with low hypomanic traits (FDR-corrected *p* > 0.05).

Kruskal–Wallis H tests examining severity grades revealed that anxiety severity was associated with discrepancies on childhood hyperactivity/impulsivity (*H* = 16.548, FDR-corrected *p* = 0.024), adult hyperactivity/impulsivity (*H* = 11.663, FDR-corrected *p* = 0.043), and childhood functional impairment (*H* = 11.610, FDR-corrected *p* = 0.043). Post hoc tests indicated that the moderate anxiety group differed significantly from the normal group on all three dimensions (FDR-corrected *p* < 0.05). In contrast, depression severity (based on SDS) showed no significant associations with any difference score after FDR correction (FDR-corrected *p* > 0.05).

### 3.3. Predictive Effects of Affective Characteristics on Self-Informant Discrepancies

#### 3.3.1. Separate Hierarchical Regressions for Each Affective Characteristic

Multicollinearity assessment indicated that variance inflation factor (VIF) values for all predictors were below 5, confirming acceptable levels of collinearity. None of the demographic variables significantly predicted self-informant discrepancies in any domain (all FDR-corrected *p* > 0.05).

Anxiety showed the most robust and consistent predictive effects, with severity positively associated with the magnitude of discrepancies (Table 6). SAS scores positively predicted discrepancies on childhood hyperactivity/impulsivity (β = 0.284, FDR-corrected *p* < 0.001), adult hyperactivity/impulsivity (β = 0.266, FDR-corrected *p* = 0.001), and childhood functional impairment (β = 0.210, FDR-corrected *p* = 0.012). Further analysis revealed that reaching the clinical threshold for anxiety (SAS ≥ 50) also significantly predicted discrepancies on childhood hyperactivity/impulsivity (β = 0.291, FDR-corrected *p* < 0.001), adult hyperactivity/impulsivity (β = 0.227, FDR-corrected *p* = 0.009), and childhood functional impairment (β = 0.186, FDR-corrected *p* = 0.042).

Depression showed more circumscribed predictive effects. SDS scores positively predicted discrepancies only on childhood hyperactivity/impulsivity (β = 0.213, FDR-corrected *p* = 0.012). Further analysis revealed that reaching the clinical threshold for depression (SDS ≥ 53) positively predicted discrepancies on adult hyperactivity/impulsivity (β = 0.181, FDR-corrected *p* = 0.045) and childhood hyperactivity/impulsivity (β = 0.174, FDR-corrected *p* = 0.045).

Hypomanic traits showed no significant predictive effects. Neither HCL-32 scores nor the presence of high hypomanic traits (HCL-32 ≥ 14) significantly predicted discrepancies in any domain (FDR-corrected *p* > 0.05).

#### 3.3.2. Combined Hierarchical Regression with All Affective Characteristics

Multicollinearity assessment indicated that variance inflation factor (VIF) values for all predictors were below 5, confirming acceptable levels of collinearity. None of the demographic variables significantly predicted discrepancies (all FDR-corrected *p* > 0.05).

In combined hierarchical regression analyses, anxiety remained the only affective characteristic with independent associations (Table 7). After controlling for demographics, depression, and hypomanic traits, SAS scores remained significantly and positively predictive of discrepancies on childhood hyperactivity/impulsivity (β = 0.269, FDR-corrected *p* = 0.027) and adult hyperactivity/impulsivity (β = 0.356, FDR-corrected *p* = 0.003). Neither depression nor hypomanic traits showed significant independent predictive effects on any domain.

Further analysis revealed that reaching the clinical threshold for anxiety (SAS ≥ 50) remained positively predictive of discrepancies on childhood hyperactivity/impulsivity (β = 0.284, FDR-corrected *p* = 0.003) and childhood functional impairment (β = 0.234, FDR-corrected *p* = 0.018). Neither the clinical threshold for depression (SDS ≥ 53) nor the presence of high hypomanic traits (HCL-32 ≥ 14) showed significant independent effects.

In addition, interaction terms (anxiety × gender and anxiety × education) were tested in the combined hierarchical regression models. None of these interactions reached statistical significance in any domain (all FDR-corrected *p* > 0.05).

#### 3.3.3. Exploratory Analysis of Anxiety Severity

To further explore the association between anxiety severity and self-informant discrepancies, hierarchical linear regression analyses were conducted focusing on the three domains for which anxiety emerged as a significant predictor in combined hierarchical regression analyses (childhood hyperactivity/impulsivity, adult hyperactivity/impulsivity, and childhood functional impairment).

After controlling for demographic variables, mild anxiety positively predicted discrepancies on childhood hyperactivity/impulsivity (β = 0.245, FDR-corrected *p* = 0.003) and adult hyperactivity/impulsivity (β = 0.175, FDR-corrected *p* = 0.034, see in Table 8). Moderate anxiety exhibited the strongest predictive effects, significantly predicting discrepancies on childhood hyperactivity/impulsivity (β = 0.276, FDR-corrected *p* < 0.001), adult hyperactivity/impulsivity (β = 0.270, FDR-corrected *p* < 0.001), and childhood functional impairment (β = 0.231, FDR-corrected *p* = 0.005). In contrast, severe anxiety did not significantly predict discrepancies in any of these domains (FDR-corrected *p* > 0.05).

## 4. Discussion

### 4.1. Sample Characteristics

This study included patients first diagnosed with ADHD in adulthood. The sample consisted primarily of young female patients (72.8%), with a mean age of 24.48 years, and a generally high level of education (77.0% held a bachelor’s degree or higher). This differs from the epidemiological characteristics observed in previous studies, where the prevalence of adult ADHD was roughly balanced between men and women [1,2].

However, this characteristic may actually reflect the clinical reality of patients first diagnosed with ADHD in adulthood. Previous research has found that female ADHD patients are prone to being underdiagnosed or misdiagnosed in childhood [5,30]; their symptoms are relatively more covert (mostly inattention) [5,31], and they often mask functional impairments with elaborate compensatory mechanisms [5,32]. Accordingly, their first clinical presentations are often delayed into adulthood, when functional compensation can no longer be sustained [5]. Furthermore, Kandeğer et al. (2025) also found that, in comparison to individuals diagnosed in childhood or adolescence, those diagnosed with ADHD in adulthood usually have higher educational attainment [22], a finding that aligns well with the compensatory mechanisms described above [2,5]. However, once patients start working or pursuing higher education, the demands on their executive function increase dramatically, and compensatory mechanisms are no longer sufficient, urging patients to seek medical advice proactively in early adulthood [23,24,25,26]. Together, these factors could explain the demographic profile of our current sample—mostly females, highly educated, and relatively young.

### 4.2. The Existence and Patterns of Self-Informant Discrepancies

This study confirmed that patient reports deviate significantly from parent reports in adults first diagnosed with ADHD in adulthood.

The generally low intraclass correlation coefficients (ICCs) between self- and parent-reports are consistent with previous studies [9,10], indicating that self-informant discrepancies are common in adults first diagnosed with ADHD in adulthood, and that relying on a single information source could introduce bias. Furthermore, analyses revealed that patients generally reported more severe symptoms and functional impairments than parents across all domains, also consistent with previous research [33].

The study also found that the magnitude of self-informant discrepancies differed by assessment content and assessment period.

Across the symptoms, self-informant consistency was higher for hyperactivity/impulsivity than for inattention. This is consistent with the “observability hypothesis” proposed in previous studies—overt symptoms (e.g., fidgeting, interrupting others) are more readily observed and measured by others, whereas covert symptoms (e.g., daydreaming) rely more on personal introspective reports [17,34]. Another recent study of adult ADHD also noted symptom observability as a factor influencing self-informant discrepancies [35].

Regarding assessment periods, self-informant consistency for childhood symptoms was generally higher than that for adulthood, with childhood hyperactivity-impulsivity showing the highest consistency and adult inattention the lowest. Two factors may explain this discrepancy: (1) parental observations are based on long-term cohabitation during childhood, whereas insight into adult behaviors relies on more limited contact [36]; and (2) the symptom profile shifts with age—overt hyperactivity/impulsivity dominates in childhood, while internalized symptoms like inattention become more prominent in adulthood, making them less observable to others [17,34].

Functional impairment showed the lowest self-informant consistency among all domains. This has important clinical consequences because functional assessment is often at the core of clinical decision-making [35]. Previous studies have suggested that the low consistency is probably due to a number of factors, including symptom implicitness, attributional differences, rater bias, and cognitive biases [37,38,39].

### 4.3. Anxiety and Self-Informant Discrepancies

The most striking finding in this study is the robust and consistent association between anxiety and self-informant discrepancies. This association persisted across multiple domains and remained independent even after controlling for depression and hypomanic traits, and did not differ significantly by gender or educational level.

#### 4.3.1. Anxiety and Hyperactivity-Impulsivity

Analysis identified anxiety as the factor robustly associated with self-informant discrepancies in the evaluation of hyperactivity-impulsivity, both in childhood and adulthood. This result may be related to the following factors. Anxiety often has somatic symptoms such as restlessness that are similar to those of ADHD, such as being hyperactive-impulsive [40]. Previous research has also reported shared neurobiology between ADHD and anxiety [41]. Thus, when anxious individuals experience these somatic symptoms, they may attribute them to ADHD, and parents may attribute them to ‘moodiness’, creating discrepancies in assessment. Furthermore, anxious individuals usually exhibit an attentional bias toward threatening information (i.e., hypervigilance) and a tendency to interpret ambiguous cues negatively [42]. This cognitive pattern may lead them to focus excessively on internalized hyperactive symptoms—such as difficulty relaxing or inner restlessness—and to evaluate these experiences more negatively, further widening self-informant discrepancies.

Further analysis revealed a positive trend between anxiety severity and the magnitude of self-informant discrepancies on hyperactivity-impulsivity. Mild and moderate anxiety were both associated with larger discrepancies, and moderate anxiety showed a stronger association. Severe anxiety, however, showed no significant association. This result may partly be due to limited statistical power, as only ten patients in the sample met the threshold for severe anxiety (based on SAS). It may also be attributed to the depletion of cognitive resources that occurs with high anxiety. Kausche et al. (2022) found that individuals with high anxiety exhibit a two-phase pattern of “early hypervigilance followed by late avoidance” in response to threatening stimuli [43]. Hypervigilance rapidly depletes cognitive resources, thus leading to avoidance processing. Under such conditions, self-informant discrepancies in highly anxious patients may not be larger than those at moderate anxiety levels. These findings should be interpreted with caution and await replication in larger samples.

#### 4.3.2. Anxiety and Childhood Functional Impairment

Anxiety was robustly positively associated with self-informant discrepancies on childhood functional impairment. This association may be related to negative memory bias.

Central cognitive features of anxiety—hypervigilance, negative interpretation, and habitual worry—may systematically distort perception of symptoms and experiences of individuals [42]. When recalling the past, these cognitive patterns may manifest as memory bias—interpreting normal childhood activities as symptomatic, and as catastrophic thinking—interpreting everyday challenges as severe functional impairment [42]. Further, research has also found that higher current emotional distress is associated with increased memory distortion [44]. Thus, when assessing childhood functional impairment, anxious patients may not evaluate the past objectively but reconstruct it under the influence of anxiety.

Notably, after controlling for depression and hypomanic traits, the independent association of SAS scores was attenuated, whereas the clinical threshold (SAS ≥ 50) was associated independently. This pattern suggests that the association may not be strictly linear. A certain level of anxiety severity may be needed before its association becomes detectable. Further analysis of anxiety severity grades supported this interpretation: moderate anxiety exhibited the strongest association with childhood functional impairment, whereas severe anxiety did not. This finding may partly be explained by limited statistical power (n = 10) or, as noted earlier, due to the depletion of cognitive resources that accompanies high anxiety states [43]. Still, given the small size of the severe anxiety subgroup, this result warrants cautious interpretation and requires further investigation in larger samples.

#### 4.3.3. Anxiety and Adult Functional Impairment

In contrast to childhood functional impairment, anxiety did not show significant independent association with self-informant discrepancies on adult functional impairment in hierarchical linear regression analyses. This finding may be attributable to the availability of more recent, objective referents in the assessment of adult functioning. These referents, such as work performance or academic records, may allow patients to calibrate their self-assessments, weakening the association between anxiety and discrepancies. By comparison, evaluations of childhood functional impairment lack such referents and may therefore be more vulnerable to the effects of anxiety [45]. Additionally, self-informant discrepancies in adult functional impairment may be associated more closely to other factors, such as reduced opportunities for parental observation and different subjective standards for evaluating daily functioning [7,9,46].

#### 4.3.4. Anxiety and Inattention

The absence of a significant independent association between anxiety and self-informant discrepancies on inattention is noteworthy. Inattention is the most implicit symptom cluster of ADHD [34], and its assessment relies heavily on introspective report. Discrepancies in this domain may instead be associated more closely to other factors, such as individual differences in introspective ability or parents’ limited opportunity to observe attention-related behaviors in their adult children [35,36,47]. These possibilities remain speculative and require further investigation.

Taken together, these findings indicate that the association between anxiety and self-informant discrepancies in adults first diagnosed with ADHD was specific to certain domains. Anxiety showed robust associations with hyperactivity-impulsivity (both childhood and adulthood) and childhood functional impairment. These patterns did not differ significantly by gender or educational level, suggesting that they were not attributable to demographic characteristics. The mechanistic accounts offered here remain speculative and require confirmation in future research.

These findings may offer several clinical implications. First, among patients with anxiety, self-informant discrepancies may be particularly pronounced for hyperactivity-impulsivity and childhood functional impairment. When encountering such discrepancies, clinicians need not presume that one report is more accurate. Rather, anxiety may serve as a clue to understanding the source of discrepancies, prompting closer scrutiny of specific behaviors. For hyperactive-impulsive symptoms, this might include follow-up questions about the frequency, context, and observable instances of the behavior. For childhood functional impairment, corroboration with objective records, such as past academic performance or teacher comments, may provide additional perspective. Second, for patients whose anxiety symptoms are prominent at the time of evaluation, this may not be the optimal moment to make clinical decisions. It may be prudent to manage the anxiety and reassess symptom presentation at a later point. Finally, this study found no independent association between anxiety and discrepancies on inattention or adult functional impairment, suggesting that discrepancies in these domains may stem from more complex sources and should not be readily attributed to anxiety.

### 4.4. Depression and Self-Informant Discrepancies

Unlike anxiety, there is no significant association between depression and self-informant discrepancies after controlling for anxiety and hypomanic traits, suggesting that its independent contribution is minimal. In separate hierarchical regression analyses for each affective characteristic, SDS scores and clinical thresholds were positively associated with discrepancies on several domains, most significantly hyperactivity-impulsivity. This is consistent with previous studies. Soble et al. (2025) reported that elevated depression scores can detect possible symptom overreporting in ADHD evaluations [16]. However, after controlling for anxiety, the associations for depression were no longer significant. This finding suggests that the apparent effects of depression in univariate analyses may be due to shared variance with anxiety. This interpretation is consistent with the moderate correlation between SDS and SAS scores observed in this sample (r=0.584) and the more robust association found for anxiety.

The relationship between depression and self-informant discrepancies may be more complex. Previous research has indicated that individuals with depression show more negative cognitive biases, including negative interpretive bias and attentional bias for negative information [15,48,49,50]. However, other research has suggested that depression may also be associated with cognitive avoidance and a tendency toward over generalized retrieval of memory, which may lead to more conservative reporting [51,52,53]. These different cognitive tendencies may be present in one person at the same time, and their relative predominance could vary across individuals. Furthermore, the association between depression and self-informant discrepancies may involve more complex mechanisms, requiring further exploration.

This study indicates that depression was not independently associated with self-informant discrepancies after controlling for anxiety. The associations observed in separate hierarchical regression analyses for each affective characteristic likely reflected shared variance with co-occurring anxiety. From a clinical perspective, when both depression and anxiety symptoms are present, anxiety may be more important when interpreting self-informant discrepancies.

### 4.5. Hypomanic Traits and Self-Informant Discrepancies

In contrast to anxiety and depression, hypomanic traits were not associated with self-informant discrepancies in this study.

Weak positive correlations emerged only in Spearman correlational analyses and were confined almost exclusively to the hyperactivity/impulsivity domain. This result may be due to symptom overlap: core features of hypomanic traits, especially thought racing and subjectively experienced accelerated thinking, are similar to hyperactivity-impulsivity symptoms of ADHD [54,55,56]. However, such experiences are highly implicit, being perceptible only to the patient and difficult for parents to observe [57]. This may lead to different attributions of symptoms. This is consistent with previous research. Barden et al. (2023) reported that among adults seeking evaluation for ADHD, self-reported ADHD and manic symptoms were highly correlated, especially on the hyperactivity-impulsivity domain [54]. The present finding that any association was restricted to this symptom cluster aligns with the overlap hypothesis.

Despite these weak correlations, hypomanic traits were not independently associated with self-informant discrepancies in hierarchical regression analyses. This is in contrast with previous studies suggesting that individuals with high hypomanic traits exhibit a positive cognitive bias, tending to overestimate their own positive qualities and evaluate their experiences more positively [20,21]. One possible explanation might be that this positive cognitive bias primarily influences the processing of positive information, whereas ADHD assessments focus on deficits. The orientation of this bias may not quite fit with the assessment content, thus could lead to the absence of a detectable association with self-informant discrepancies.

Overall, the present findings do not support hypomanic traits as an independent factor associated with self-informant discrepancies among adults first diagnosed with ADHD in adulthood. In clinical practice, clinicians should avoid uncritically attributing self-informant discrepancies to hypomanic traits.

### 4.6. Limitations and Future Directions

This study has the following limitations, which should be taken into account when interpreting the findings.

First, this study focused on adults first diagnosed with ADHD in adulthood. The sample comprised predominantly young females (72.8%) with high educational attainment (77.0% bachelor’s degree or higher). In regression analyses, none of the demographic variables significantly predicted self-informant discrepancies. Moderation analyses further indicated that the association between anxiety and discrepancies did not differ significantly by gender or educational level. These findings suggest that the demographic characteristics of the sample are unlikely to have substantially confounded the main conclusions. Nevertheless, given the specific demographic profile of the sample, caution remains warranted when generalizing these findings to broader populations.

Second, the cross-sectional design excludes causal conclusions about the associations between affective characteristics and self-informant discrepancies. Future studies are needed to identify the direction of these associations and to examine potential underlying mechanisms.

Third, although parental familiarity was not independently associated with self-informant discrepancies in this study, parent reports could still be biased [57]. Parents may have limited insight into their adult children’s daily functioning, and they could also underestimate functional impairment or symptom severity [58,59]. Thus, this study only examined associations between affective characteristics and self-informant discrepancies, rather than determining which source is more accurate. Future studies could include other informants, such as spouses, to evaluate patterns of discrepancies across different informant types and to better evaluate potential sources of parental reporting bias.

Fourth, subgroups of patients with severe anxiety (n = 10) and severe depression (n = 28) were small, which may have reduced the statistical power for detecting effects at these severity levels. Therefore, results involving anxiety and depression severity grades should be interpreted with caution and need to be tested in larger samples.

Finally, this study did not include measures that detect deliberate symptom exaggeration. Although the diagnostic assessment involved confirmation by multiple experienced clinicians, therefore alleviating concerns of malingering, the possibility of intentional overreporting could not be fully excluded. Previous research has shown that overreporting occurs in ADHD evaluations [59,60,61] and may contribute to self-informant discrepancies. Future studies could include symptom validity indicators to help detect intentional overreporting.

## 5. Conclusions

This study examined the association between anxiety, depression, hypomanic traits and self-informant discrepancies across symptom and functional impairment domains among adults first diagnosed with ADHD in adulthood.

Among the affective characteristics examined, anxiety showed the most robust and consistent pattern of association. Anxiety was independently associated with greater discrepancies on hyperactivity-impulsivity (both childhood and adulthood) and childhood functional impairment. Depression, by contrast, did not show independent associations with discrepancies after controlling for anxiety. Hypomanic traits were not independently associated with discrepancies in any analysis.

These findings suggest that, when evaluating adults first diagnosed with ADHD in adulthood, clinicians should attend to the patient’s current anxiety level when interpreting self-informant discrepancies. Anxiety may serve as a clue to understanding the source of self-informant discrepancies. Given the cross-sectional design and the specific demographic profile of the sample, future research with more diverse samples and varied methodologies is needed to further clarify the direction of these associations and to explore potential underlying mechanisms.

## Figures and Tables

**Table 1:** Demographic and clinical characteristics of the sample (N = 217).

	Category	n (%)	Mean ± SD	Range
Age (years)			24.48 ± 4.00	18–37
Gender	Male	59 (27.2)		
	Female	158 (72.8)		
Education	Junior high or below	24 (11.1)		
	Vocational college	26 (12.0)		
	Bachelor’s degree	139 (64.1)		
	Master’s or above	28 (12.9)		
Parental Familiarity	Less familiar	32 (14.7)		
	Familiar	98 (45.2)		
	Very familiar	87 (40.1)		
Depression (SDS)	Standard score		59.18 ± 10.87	
	Below threshold (<53)	61 (28.1)		
	At or above threshold (≥53)	156 (71.9)		
	Severity			
	Mild (53–62)	78 (35.9)		
	Moderate (63–72)	50 (23.0)		
	Severe (≥73)	28 (12.9)		
Anxiety (SAS)	Standard score		51.48 ± 10.42	
	Below threshold (<50)	92 (42.4)		
	At or above threshold (≥50)	125 (57.6)		
	Severity			
	Mild (50–59)	69 (31.8)		
	Moderate (60–69)	46 (21.2)		
	Severe (≥70)	10 (4.6)		
Hypomanic Traits (HCL-32)	Total score		16.72 ± 6.24	
	Low hypomanic traits (<14)	59 (27.2)		
	High hypomanic traits (≥14)	158 (72.8)		

Note: SDS, Self-Rating Depression Scale; SAS, Self-Rating Anxiety Scale; HCL-32, 32-item Hypomania Checklist. Data are presented as n (%), or Mean (SD). Severity classifications are based on scale scores (Chinese norms) and reflect symptom severity rather than clinical diagnoses. As noted in Section 2.3.2, terms such as “depression,” “anxiety,” and “hypomanic traits” are used throughout for brevity to refer to scale-based groupings or continuous scores.

**Table 2:** Self- and informant-reported scores on different domains: differences and consistency analysis.

Domains	Self-Report (M ± SD)	Informant-Report (M ± SD)	Mean Difference	Cohen’s d	Wilcoxon Z	ICC (95% CI)
Childhood inattention	7.06 ± 2.23	3.72 ± 2.99	3.34	0.86	−10.21	0.151 (−0.050, 0.338)
Adult inattention	7.82 ± 1.56	3.66 ± 3.12	4.16	0.92	−12.55	0.066 (−0.048, 0.190)
Childhood hyperactivity/impulsivity	4.03 ± 2.78	1.82 ± 2.31	2.21	0.62	−8.45	0.290 (0.027, 0.491)
Adult hyperactivity/impulsivity	4.26 ± 2.47	1.80 ± 2.08	2.46	0.74	−9.87	0.222 (−0.032, 0.433)
Childhood functional impairment	3.35 ± 1.48	1.35 ± 1.64	2.00	0.76	−9.12	0.075 (−0.041, 0.194)
Adult functional impairment	4.21 ± 1.11	1.88 ± 1.96	2.34	0.84	−10.98	0.062 (−0.043, 0.173)

Note: ICC, intraclass correlation coefficient; CI, confidence interval. All paired comparisons were significant at FDR-corrected *p* < 0.001. ICC values are presented with 95% confidence intervals in parentheses. Cohen’s d effect sizes: 0.2 small, 0.5 medium, 0.8 large.

**Table 3:** Spearman correlations between continuous affective characteristics variables and self-informant discrepancies.

Affective Characteristics	Domain	r_s_	Corrected *p*
Anxiety (SAS)	Childhood hyperactivity/impulsivity	0.282	0.009
	Adult hyperactivity/impulsivity	0.251	0.005
	Childhood functional impairment	0.223	0.005
	Adult inattention	0.163	0.031
	Adult functional impairment	0.154	0.038
Depression (SDS)	Childhood hyperactivity/impulsivity	0.233	0.006
	Adult inattention	0.182	0.025
	Childhood inattention	0.174	0.026
	Childhood functional impairment	0.165	0.030
	Adult hyperactivity/impulsivity	0.150	0.041
Hypomanic traits (HCL-32)	Adult hyperactivity/impulsivity	0.177	0.027
	Childhood hyperactivity/impulsivity	0.166	0.032

Note. Only significant correlations after FDR correction are shown (FDR-corrected *p* < 0.05).

**Table 4:** Spearman correlations between categorial affective characteristics variables and self-informant discrepancies.

Affective Characteristics	Categorial Variable	Domain	r_s_	Corrected *p*
Anxiety (SAS)	Clinical threshold (≥50)	Childhood hyperactivity/impulsivity	0.267	0.015
	Severity grades	Childhood hyperactivity/impulsivity	0.272	0.008
		Childhood functional impairment	0.230	0.010

Note. Only significant correlations after FDR correction are shown (FDR-corrected *p* < 0.05).

**Table 5:** Group comparison of self-informant discrepancies based on affective characteristics.

Affective Characteristics	Domain	Group/Severity	Test Statistic	Corrected *p* (FDR)	Direction
Anxiety (SAS)	Childhood hyperactivity/impulsivity	Below vs. at/above threshold	U=3968.0,Z=−3.931	0.024	Anxiety > non-anxiety
	Adult hyperactivity/impulsivity	Below vs. at/above threshold	U=4435.5,Z=−2.898	0.024	Anxiety > non-anxiety
	Childhood functional impairment	Below vs. at/above threshold	U=4405.5,Z=−2.974	0.024	Anxiety > non-anxiety
	Adult functional impairment	Below vs. at/above threshold	U=4621.0,Z=−2.503	0.048	Anxiety > non-anxiety
	Childhood hyperactivity/impulsivity	Severity (Kruskal–Wallis)	H=16.548	0.024	Moderate > normal
	Adult hyperactivity/impulsivity	Severity (Kruskal–Wallis)	H=11.663	0.043	Moderate > normal
	Childhood functional impairment	Severity (Kruskal–Wallis)	H=11.610	0.043	Moderate > normal

Note. *p*-values were corrected for multiple comparisons using the Benjamini–Hochberg false discovery rate (FDR) method. Only comparisons with corrected *p* < 0.05 are shown.

**Table 6:** Separate hierarchical linear regression for each affective characteristic.

Dependent Variable	Significant Predictor	Predictor Type	β	Corrected *p*
Childhood hyperactivity/impulsivity	Anxiety (SAS standardized scores)	Continuous	0.284	<0.001
Adult hyperactivity/impulsivity	Anxiety (SAS standardized scores)	Continuous	0.266	0.001
Childhood functional impairment	Anxiety (SAS standardized scores)	Continuous	0.210	0.012
Childhood hyperactivity/impulsivity	Depression (SDS standardized scores)	Continuous	0.213	0.012
Childhood hyperactivity/impulsivity	Anxiety threshold (SAS ≥ 50)	Dichotomous	0.291	<0.001
Adult hyperactivity/impulsivity	Anxiety threshold (SAS ≥ 50)	Dichotomous	0.227	0.009
Childhood functional impairment	Anxiety threshold (SAS ≥ 50)	Dichotomous	0.186	0.042
Adult hyperactivity/impulsivity	Depression threshold (SDS ≥ 53)	Dichotomous	0.181	0.045
Childhood hyperactivity/impulsivity	Depression threshold (SDS ≥ 53)	Dichotomous	0.174	0.045

Note. All models controlled for demographic variables (gender, age, education, and informant familiarity) in Block 1, with each affective characteristic entered separately in Block 2. Only significant predictors after Benjamini–Hochberg FDR correction (FDR-corrected *p* < 0.05) are shown.

**Table 7:** Combined hierarchical linear regression with all affective characteristics.

Dependent Variable	Significant Predictor	Predictor Type	β	Corrected *p*
Childhood hyperactivity/impulsivity	Anxiety (SAS)	Continuous	0.269	0.027
Adult hyperactivity/impulsivity	Anxiety (SAS)	Continuous	0.356	0.003
Childhood hyperactivity/impulsivity	Anxiety threshold (SAS ≥ 50)	Dichotomous	0.284	0.003
Childhood functional impairment	Anxiety threshold (SAS ≥ 50)	Dichotomous	0.234	0.018

Note. Only significant predictors after Benjamini–Hochberg FDR correction (FDR-corrected *p* < 0.05) are shown.

**Table 8:** Hierarchical linear regression for anxiety severity grades predicting self-informant discrepancies.

Severity of Anxiety	Domain	β	Corrected *p*
Mild	Childhood hyperactivity/impulsivity	0.245	0.003
	Adult hyperactivity/impulsivity	0.175	0.034
	Childhood functional impairment	—	>0.05
Moderate	Childhood hyperactivity/impulsivity	0.276	<0.001
	Adult hyperactivity/impulsivity	0.270	<0.001
	Childhood functional impairment	0.231	0.005
Severe	All domains	—	>0.05

Note. All models controlled for demographic variables (gender, age, education, parental familiarity). *p*-values were corrected using the Benjamini–Hochberg FDR method. Dashes indicate non-significant results after correction (corrected *p* > 0.05).

## Data Availability

The data presented in this study are available on request from the corresponding author due to the sensitive nature of the clinical information, which cannot be made publicly accessible to protect participant privacy.

## Abbreviations

The following abbreviations are used in this manuscript:

ADHD	Attention deficit hyperactivity disorder
DIVA-5	The Diagnostic Interview for Adult ADHD(newest version)
SDS	The Self-Rating Depression Scale
SAS	The Self-Rating Anxiety Scale
HCL-32	The 32-item Hypomania Checklist
